# Damnable!

**Published:** 1920-04-10

**Authors:** 


### "Damnable!"

A deplorable example of some consequences of the housing problem was related to the City Coroner, Dr. F. J. Waldo, a short time ago at an inquest on a woman who had died in Southwark, amid squalid surroundings, from chronic heart trouble accelerated by infestation with lice. The story was that the woman lived with her husband and three children in two rooms; five other families occupied ten rooms on the same floor, and each of the other two floors accommodated six families, as well as a large number of cats. The daughter of the woman said the conditions under which they lived were terrible, but they were unable to find other accommodation. The rooms were very dark and damp. The brother of the woman said he was greatly upset when he found his sister living in such a place; he had never seen anything like it, and had had to light his pipe to guard against infection. The place was unfit for human habitation.

The Coroner pointed out the danger from such conditions of outbreaks of typhus (a filth disease), measles (one of the most deadly maladies amongst the children in poor, crowded districts), smallpox (against which so many more are now unprotected by vaccination), and he remarked that the whole of the Metropolis might be affected as a result. It is a scandal that children should have to be shut up with cats, who are liable to spread diphtheria and other diseases. At least, the majority of the mangy cats might be exterminated. The Coroner recalled the fact that the Prince of Wales had described the conditions under which people lived in the slum areas of Southwark as "damnable." This is putting it mildly.

